# Prevalence, virulence and antifungal activity of *C. albicans* isolated from infected root canals

**DOI:** 10.1186/s12903-020-01347-5

**Published:** 2020-12-01

**Authors:** Sheela B. Abraham, Farah al Marzooq, Wan Harun Himratul-Aznita, Hany Mohamed Aly Ahmed, Lakshman Perera Samaranayake

**Affiliations:** 1 Department of Preventive and Restorative Dentistry, College of Dental Medicine, University of Sharjah, Sharjah, UAE; 2 Department of Medical Microbiology and Immunology, College of Medicine and Health Sciences, United Arab Emirates University, Al Ain, UAE; 3 Department of Oral and Craniofacial Sciences, Faculty of Dentistry, University of Malaya, Kuala Lumpur, Malaysia; 4 Department of Restorative Dentistry, Faculty of Dentistry, University of Malaya, Kuala Lumpur, Malaysia; 5 Department of Oral and Craniofacial Health Sciences, College of Dental Medicine, University of Sharjah, Sharjah, UAE; 6 Faculty of Dentistry, University of Hong Kong, Pok Fu Lam, Hong Kong

**Keywords:** *Candida albicans*, Root canal infection, Virulence, Haemolysin, Phospholipase, Antifungals, Biofilm formation

## Abstract

**Background:**

There is limited data on the prevalence of *Candida* species in infected root canal systems of human teeth. We attempted to investigate the prevalence, genotype, virulence and the antifungal susceptibility of *Candida albicans* isolated from infected root canals of patients with primary and post-treatment infections in a UAE population.

**Methods:**

Microbiological samples from 71 subjects with infected root canals were aseptically collected, and cultured on Sabouraud dextrose agar, and *C. albicans* was identified using multiplex polymerase chain reaction, and the isolates were further subtyped using ABC genotyping system. Their relative virulence was compared using further four archival samples of endodontic origin from another geographical region, and four more salivary isolates, as controls. The virulence attributes compared were biofilm formation, and production of phospholipase and haemolysin, and the susceptibility to nystatin, amphotericin B, ketoconazole, and fluoconazole was also tested.

**Results:**

4 out of 71 samples (5.6%) yielded *Candida* species. On analysis of variance among the groups, the intracanal isolates, mainly Genotype A, possessed a high degree of phospholipase and haemolysin activity (*p* < 0.05). The UAE and Finland isolates were stronger biofilm formers and had superior phospholipase production capacity compared with the salivary isolates. All isolates were sensitive to the antifungal chemicals used. The salivary isolates were more sensitive to fluoconazole compared to other groups (*p* < 0.05).

**Conclusion:**

The prevalence of *C. albicans* in infected root canals of patients attending a dental hospital in UAE is low. The strains isolated were good biofilm formers, possessed strong phospholipase and haemolysin activity and were mostly of the genotype A. The fact that the root canal isolates possessed significant hydrolase activity, imply that they are equipped with virulence attributes necessary for survival within a harsh intracanal ecosystem.

## Background

Although the contribution of bacteria to the pathogenesis of endodontic infections has been well established [1], the role of other microorganisms such as fungi [2], viruses [3], bacteriophages [4] and archaea [5] in the disease process is unclear. Fungal eukaryotes comprise a small part of the oral microbiome, of which the largest proportion is made up of *Candida* species with a predominance of *C. albicans* [6]. The latter is the most potent of the *Candida* species due to its superior virulence attributes such as tenacious adhesion to host surfaces, and subsequent biofilm formation, production of hydrolytic enzymes, phospholipases, haemolysins and proteinases, and phenotypic switching [7]. Hence, *C. albicans*, in comparison to its counterparts, appear to possess superior environmental adaptability, to evade the host defenses in adverse habitats such as the endodontic ecosystems.

The portal of entry of yeasts into an infected endodontic eco-niche is likely to be through cracks and leakage around faulty restorations. They may also enter through dentinal tubules of deep caries lesions and invade frank pulpal exposures [8]. The biofilm lifestyle of *Candida,* which confers intrinsic resistance to antifungal chemicals [9], enables these eukaryotes to withstand the iatrogenic chemical assaults and persist within the root canal system causing chronic infections even after root canal therapy. It has also been proposed that after *C. albicans* adheres to the host cells, the hydrolases that are secreted by their hyphae allow penetration into the host cells [10].The secreted hydrolases are also thought to enhance the efficiency of acquiring extracellular nutrients [11], thereby contributing to yeast survival in the endodontic ecosystem.

Multiple studies evaluating the mycobiome in various oral and other anatomical eco-niches of differing population groups in diverse geographic locale have shown the ubiquity of *C. albicans* [12–14]. On the contrary, there is limited data on the prevalence of *Candida* species in infected root canal systems of human teeth. Furthermore, Samaranayake [15] has shown that the oral prevalence of *Candida* varies in different geographic locales even in healthy population groups. There is no information, to the best of our knowledge, on the prevalence and virulence attributes of *Candida* species in infected root canals from patients in Asia or the Middle East. The objectives of this study were first, to determine the prevalence of *C. albicans* in infected root canals of patients, with either primary or post-treatment endodontic infections attending the University Dental Hospital Sharjah (UDHS), UAE (United Arab Emirates). The second objective was to characterize and genotype the isolated strains and compare their virulence properties and antifungal susceptibility with endodontic isolates from another geographical region (Finland), and salivary isolates from healthy individuals (the reference control group).

## Methods

### Study subjects and informed consent

The Research and Ethics committee of the University of Sharjah approved the protocol (REC No. 18-05-30-01) for this study and informed consent was obtained from each patient. Patients who attended the endodontic clinic at the UDHS were invited to participate in the study. The patients were grouped as follows: Group 1: Irreversible pulpitis free of PA (periapical) involvement (n = 18). They were those who experienced spontaneous pain or long lasting moderate to severe pain during cold test with Endo-Ice (1,1,1,2 tetrafluoroethane; Hygienic Corp, Akron, OH) and a bleeding coronal pulp on access opening.

Group 2: Irreversible pulpitis with PA involvement or symptomatic apical periodontitis (n = 22), These patients presented with inflammation of the apical periodontium which exhibited a painful response to biting, percussion or palpation, with or without an apical radiolucent area. The radiograph exhibited at least a widened periodontal ligament space [16]. Group 3: Post-treatment infections scheduled for retreatment (n = 21); subgroup 1-symptomatic (n = 14), subgroup 2-asymptomatic (n = 7), Group 4: Control (n = 3) (cases scheduled for elective root canal treatment). These were patients that had healthy teeth but were indicated for intentional root canal treatment as the tooth was an abutment in a prosthodontic treatment plan.

Patients in whom rubber dam could not be applied, patients with generalized periodontal disease with edematous or fibrotic gingiva, periodontal pockets more than 3 mm, horizontal or vertical bone loss, advanced mobility, presence of intra canal posts and radicular fractures were excluded from the study. Patients with systemic diseases, cancer, immunodeficiency disorder and patients on antibiotics three months prior to sampling were also excluded.

For post-treatment cases, adult patients with a root canal treated tooth, done at least 1 year ago showing radiographic evidence of apical periodontitis were included. The following findings were recorded: gender, age, tooth type, clinical symptoms, tenderness to percussion, presence/absence of periapical radiolucency and the radiographic quality of the root-canal filling. Coronal restorations were categorized as defective if open margins, fractured restorations or recurrent decay was present.

### Sample collection

The protocol for sample collection was that described by Pinheiro et al. [17]. After rubber dam isolation, the tooth and the surrounding field were cleansed with 3% hydrogen peroxide and disinfected with a 2.5% sodium hypochlorite (NaOCl) solution. For primary infected teeth, the pulp was accessed with sterile high-speed carbide burs. Following this, the access cavity of the tooth, clamp, and adjacent rubber dam was again disinfected with 2.5% NaOCl. The solution was inactivated with 5% sodium thiosulphate in order to avoid contamination. Sampling and culture were performed later to check the effectiveness of the isolation following thorough disinfection of the area around the tooth to ensure sterility and avoiding contamination of the sample obtained. Pulp was extirpated and sterile saline was used for intracanal irrigation.

A hand file was then inserted, up to 1 mm short of the radiographic root apex with a gentle filing motion, following which two #25 paper points were inserted to the same level and used to soak up the fluid in the canal, and kept in place for a minute. A single root canal was always sampled in order to confine the microbial evaluation to a single ecological environment. In multi-rooted teeth, the root with the periapical lesion was selected. If all roots had periapical lesions, the widest canal was sampled.

For post-treatment infection cases, using the same sterile protocol, the gutta percha was removed by sterile Gates Glidden burs #3 and #4 (Dentsply Sirona Endodontics, USA) and the Protaper retreatment files, sizes D1, D2 and D3(Dentsply Sirona Endodontics,). The apical material was retrieved using #25 K-type or Hedström files (Dentsply Sirona Endodontics). No solvents were used for removal of the gutta percha. After gutta percha removal, samples were collected with paper points as mentioned above.

Using a strict aseptic technique, the paper points were collected by a single trained operator (SA) and inserted in two 1.5 ml sterile tubes; the first tube contained 300 µl of Phosphate Buffered Saline (PBS) for the DNA extraction and further molecular tests, while the second tube contained 300 µl of brain–heart infusion (BHI) broth (Thermo Fisher Scientific Remel, USA) to be used for yeast culture. Negative control using sterile paper points, not applied to the root canal was used in parallel to test for contaminants in the paper points. The samples were taken to the laboratory for processing within 2–4 h of collection.

### Microbial culture

In the laboratory, the tube containing the sample in 0.3 ml of BHI broth was cultured aerobically on Sabouraud dextrose agar (SDA) (Himedia, India) at 37 °C for 48 h, and the resultant growth, if any, was harvested and pure colonies obtained after subculture for 24 h on Sabouraud agar were then stored in 20% glycerol.

A total of four endodontic isolates of *C. albicans* from another geographical region (Finland) were used for comparative purposes. These were archival strains kindly donated by Dr. TMT Waltimo to the Oral and Biosciences Laboratory, Faculty of Dentistry, Hong Kong University, Hong Kong) [18]. These isolates were obtained from subjects with primary endodontic infection who were systemically healthy.

Saliva samples from healthy subjects in the UAE were collected randomly to isolate *C. albicans* for use as reference, control strains of non-endodontic origin, from the oral commensals. For this purpose, saliva from each volunteer was collected by expectoration into a 10 mL container, and cultured in the laboratory as described above, to obtain pure samples, and stored until use in the experiments. The salivary samples were processed following a protocol of Samaranayake et al. [19].

### Phenotypic identification

Pure colonies from Sabouraud dextrose agar were used for the germ tube test [20] wherein, an isolated fungal colony of *C. albicans* was incubated with Fetal bovine serum (FBS) (Himedia, India) at 37 °C for 2 to 4 h, and viewed microscopically for germ tube growth [1].

The isolates were also grown on CHROMagar (HiCrome TM Candida Differential Agar, M1297A (Himedia, India) for the chromogenic confirmation of the isolates identified as *C. albicans* which produce pure light green colonies.

### Genotypic identification

#### Confirmation of fungal isolates identified as *C. albicans* by DNA isolation and Multiplex PCR

The second aliquot in PBS from yeast positive clinical samples was subjected to DNA isolation. DNA extraction was performed using MasterPureTM Complete DNA and RNA Purification (Epicenter, USA), following manufacturer’s guidelines. The quality and quantity of the extracted DNA was assessed using a Colibri Microvolume Spectrometer (Titertek-Berthold Detection Systems GmbH, Germany). DNA samples were considered pure if the A260/280 ratio were more than 1.8, and A260/230 values were in the range of 1–2.2.

Confirmation of the fungal isolates identified as *C. albicans* was done using multiplex PCR (polymerase chain reaction) according to the method of Trost et al. [21], with minor modifications. The Multiplex PCR was based on the amplification of two fragments from the Candida ITS1 and ITS2 regions by the combination of two-yeast-specific and six-species specific primers in a single PCR reaction (Table 1). PCR was performed under the following cycling conditions: 40 cycles of 15 s at 94 °C, then 30 s at 55 °C, and 45 s at 65 °C, after a 10-min initial period of DNA denaturation and enzyme activation at 94 °C. All PCR-reaction products were evaluated by electrophoresis in 2.0% (w/v) agarose gels run at 90 V for 60 min. In total, *C. albicans* isolates were identified using three different methods; germ tube test, culture on Chromagar media and finally Multiplex PCR.

**Table 1:** Amplicon sizes (base pairs) results from Multiplex PCR amplification using yeast specific (Universal-UNI1 and UNI2) and corresponding species-specific primers of *Candida* species

Species	Primer	Sequence (5′-3′)	Amplicon size (bp)
	UNI 1		
	UNI 2		
*C. albicans*	Calb	AGCTGCCGCCAGAGGTCTAA	583/446
*C. tropicalis*	Ctro	GATTTGCTTAATTGCCCCAC	583/507
*C. krusei*	Ckru	CTGGCCGAGCGAACTAGACT	590/169
*C. glabrata*	Cgla	TTGTCTGAGCTCGGAGAGAG	929/839
*C. dubliniensis*	Cdub	CTCAAACCCCTAGGGTTTGG	591/217
*C. parapsilosis*	Cpar	GTCAACCGATTATTTAATAG	570/370

#### ABC genotyping

We aimed to investigate the genotypic distribution of *C. albicans* isolates and examine whether there was an association between these genotypes and their virulence properties. We followed the method developed by McCullough et al.[22] which uses a pair of PCR primers designed to span the region that includes the site of the transposable group I intron of the 25S rRNA gene (rDNA), and can classify *C. albicans* strains into three genotypes based on the amplified PCR product length: genotype A (approximately 450 bp product), genotype B (approximately 840 bp product), and genotype C (approximately 450 and 840 bp products). ABC genotyping was performed with the following primers: (5′ATAAGGGAAGTCGG-CAAAATAGATCCGTAA-3′) and (CCTTGGCTGTGGTTTCGCTAGATAGTAGAT-3′) using PCR protocol and parameters reported previously. All PCR reaction products were evaluated by electrophoresis in 2% (w/v) in agarose gels run at 90 V for 60 min.

### Virulence assays

A total of 12 isolates, eight endodontic isolates, four each from UAE and Finland, and four salivary isolates from UAE were used in the following virulence studies. Each experiment was conducted on three separate occasions, and the mean value calculated.

#### Evaluation of the biofilm formation

The isolates were tested for biofilm formation by the method of Jin et al. [23], with modification. A suspension of 10^3^ cells/ml was prepared in Roswell-Park Memorial Institute (RPMI) 1640 medium w/l-glutamine, 0.2% glucose, and 0.165 mol/l MOPS buffer w/o sodium bicarbonate (AT180, RPMI-1640, Sigma-Aldrich, USA). RPMI 1640 was supplemented with 2% d-glucose. Flat bottom 96-well microtiter plates (Corning, 3370 Polypropylene, Sigma-Aldrich, USA) were used. The plates with the fungal suspensions (200 µl) were then incubated at 37 °C for 24 h in a shaking incubator (Thermo Fisher Scientific4430, USA) at 90 rpm. Unattached fungal cells were carefully aspirated using multichannel pipette without disrupting the biofilms and fresh RPMI media was added to all the wells. The plates were then incubated in static position overnight. After biofilm formation at 24 h, the medium was carefully aspirated using multichannel pipette without disrupting the biofilms. The plates were washed thrice with sterile PBS (200 μl/well) and were drained in an inverted position by blotting with a paper towel after the last wash, to remove any residual RPMI. After this 1% solution of crystal violet (CV) (100 µL each well) was added to each well for 15 min, then washed three times with water and dried at room temperature. Optical density (OD) of stained adherent biofilm was obtained by using micro ELISA autoreader (model 680, Biorad, UK) at wavelength 570 nm.

The quantitation of biofilms based on metabolic activity was later performed by the XTT (2,3-bis[2-methoxy-4-nitro-5-sulfo-phenyl]-2H-tetrazolium-5-carboxanilide) reduction assay. The assay is based on the cleavage of the yellow tetrazolium salt XTT to form an orange formazan dye, which may occur exclusively by means of viable cells. The increase in the formazan dye production (measurable spectrophotometrically at 490 nm) is proportional to the effective number of live biomass. Briefly, 100 μl of XTT/menadione solution (Sigma-Aldrich, USA) was added to each well containing pre-washed 24 h biofilm. The plates were then incubated for 2 h at 37 °C, after which 80 μl of the resultant coloured supernatant from each well was then transferred to a new microtiter plate, and optical densities (OD) at 492 nm were read with an ELISA plate reader (Bio-Rad, UK). The experiments were performed in triplicate for each isolate. The interpretation of the results requires defining the cut-off value (ODc) which is obtained from 3 times the standard deviation of the negative. When OD ≤ ODc, it is a non-biofilm former, OD ≤ 2 ODc-weak, OD between 2 ODc-4 ODc-moderate and when OD ≥ 4 ODc-strong biofilm former [24].

#### Phospholipase assay

Candida isolates were tested for phospholipase activity on egg-yolk agar, by the method described by Samaranayake et al. [25]. The egg-yolk medium comprising 13 g SDA, 0.11 g CaCl2, 11.7 g NaCl, and 10% egg-yolk emulsion was prepared. A suspension of 10 μl of yeast cells which was adjusted to 10^8^ cells/ml were inoculated on an egg-yolk agar and left to dry at room temperature. Measurement of the zone of precipitation, considered as positive phospholipase activity (Pz) was conducted according to the method explained by Price et al. [26]. After four days of incubation, measurements of colony diameter and colony diameter plus precipitation zone of each isolate were made. Phospholipase activity was expressed as Pz and calculated by the ratio of the colony diameter to the diameter of the colony plus precipitation zone expressed in millimeter (mm). Based on this, phospholipase (Pz) activities were classified as follows: Weak = 0.90–0.99 mm, Mild: 0.80–0.89 mm, Moderate: 0.70–0.79 mm, Strong: Pz < 0.69 mm. *C. albicans* (ATCC 90,028) and *C. parapsilosis* (ATCC 22,019) were used as controls. In this assay, the higher the Pz value, the lower was the phospholipase activity.

#### Haemolysin assay

Haemolysin activity (Hz) of *C. albicans* was detected using the modified method described by Sachin et al. [27] with modifications using SDA supplemented with 3% glucose and human blood (pH = 5.6). Cell suspensions of test and control strains containing 10^8^ cells/ml were deposited on the agar and allowed to dry. Plates were incubated at 37 °C for 48 h in 5% CO_2_. *C. albicans* (ATCC 90028) and *C. parapsilosis* (ATCC 22019) were used as positive and negative controls respectively. A positive haemolytic activity was indicated by the presence of a zone of haemolysis around colonies viewed with transmitted light. After 48 h incubation, diameter of both the translucent zone surrounding the colony and the diameter of the colony were measured. Haemolytic activity (Hz) was calculated as the ratio of the diameter of the colony to that of the translucent zone of haemolysis expressed in millimeter (mm). Based on this, haemolysin (Hz) activities were classified as Weak: 0.90–0.99 mm, Mild: 0.80–0.89 mm, Moderate: 0.70–0.79 mm Strong: Hz < 0.69 mm.

### Antifungal sensitivity

The 12 isolates were tested for in vitro susceptibility to antifungals amphotericin B, fluoconazole, ketoconazole and nystatin using the CLSI broth microdilution methods as outlined in document M27-A3 [28] using 96 well microtiter plates (Corning, 3370 Polypropylene flat bottom 96 well). Briefly, a yeast suspension of 10^6^ cells/mL from a 24-h old culture was grown in Sabouraud Dextrose broth (ME033, Himedia) and adjusted to 0.5 McFarland standard using a densitometer (Grant Instruments TM Grant Bio TM Densitometer). Amphotericin B (Sigma Aldrich, USA) and Ketoconazole (Sigma-Aldrich) were prepared by dissolving in 5% dimethylsulphoxide (DMSO) and Fluoconazole (Sigma-Aldrich) was prepared in sterile distilled water. Nystatin (Sigma-Aldrich), a polyene was dissolved in DMSO and absolute ethanol (3:2 ratio), respectively, and was prepared initially as 10,000 mg/ml solutions and stored at − 20 °C before use. It was suspended/diluted in RPMI 1640 medium, buffered with 0.165 M MOPS [3-(N-morpholino) propanesulfonic acid] containing l-glutamine and lacking sodium bicarbonate (Sigma-Aldrich) and incubated at 35 °C. Minimum inhibitory Concentration (MIC) values were determined visually after 24 h of incubation, as the lowest concentration of drug which caused a significant reduction of growth below control levels. Two quality control strains were used; *C. albicans* ATCC 90028 and *C. parapsilosis* ATCC 22019. All tests were replicated on three separate occasions to ensure accuracy and reproducibility of experiments.

Data distributions were expressed as means and standard deviations (SD). The numerical data were analyzed using analysis of variance (ANOVA) to compare the results among the three C*andida* groups (UAE, Finland and Saliva), and the significant difference within groups was analyzed using the Bonferroni test. Data were analyzed using Statistical Package for Social Sciences version 22 (SPSS Inc., Chicago, IL, USA). All results were considered significant at *p* < 0.05.

## Results

### Yeast prevalence in UAE endodontic samples

Samples were collected from 71 participants; 57 males and 14 females, and their ages ranged between 18 and 60 years. Yeast growth was obtained only from 4 of 71 samples (5.6%). Of the 4 strains obtained, one isolate was from the clinical group 2 (primary infection; irreversible pulpitis with symptomatic apical periodontitis) and the remainder from group 3 (post treatment infection; subgroup 1-one isolate and subgroup 2-two isolates.

All 12 *C. albicans* strains from UAE (four strains) and Finland (four strains) and saliva (four strains) were evaluated in order to compare their genotypes, virulence properties and antifungal susceptibility.

#### Genotypes

All four isolates from UAE belonged to genotype A, compared to Finnish isolates which comprised three Type A, and one Type B and the saliva group contained one each of Type A and B and two of genotype D.

**Fig. 1:**
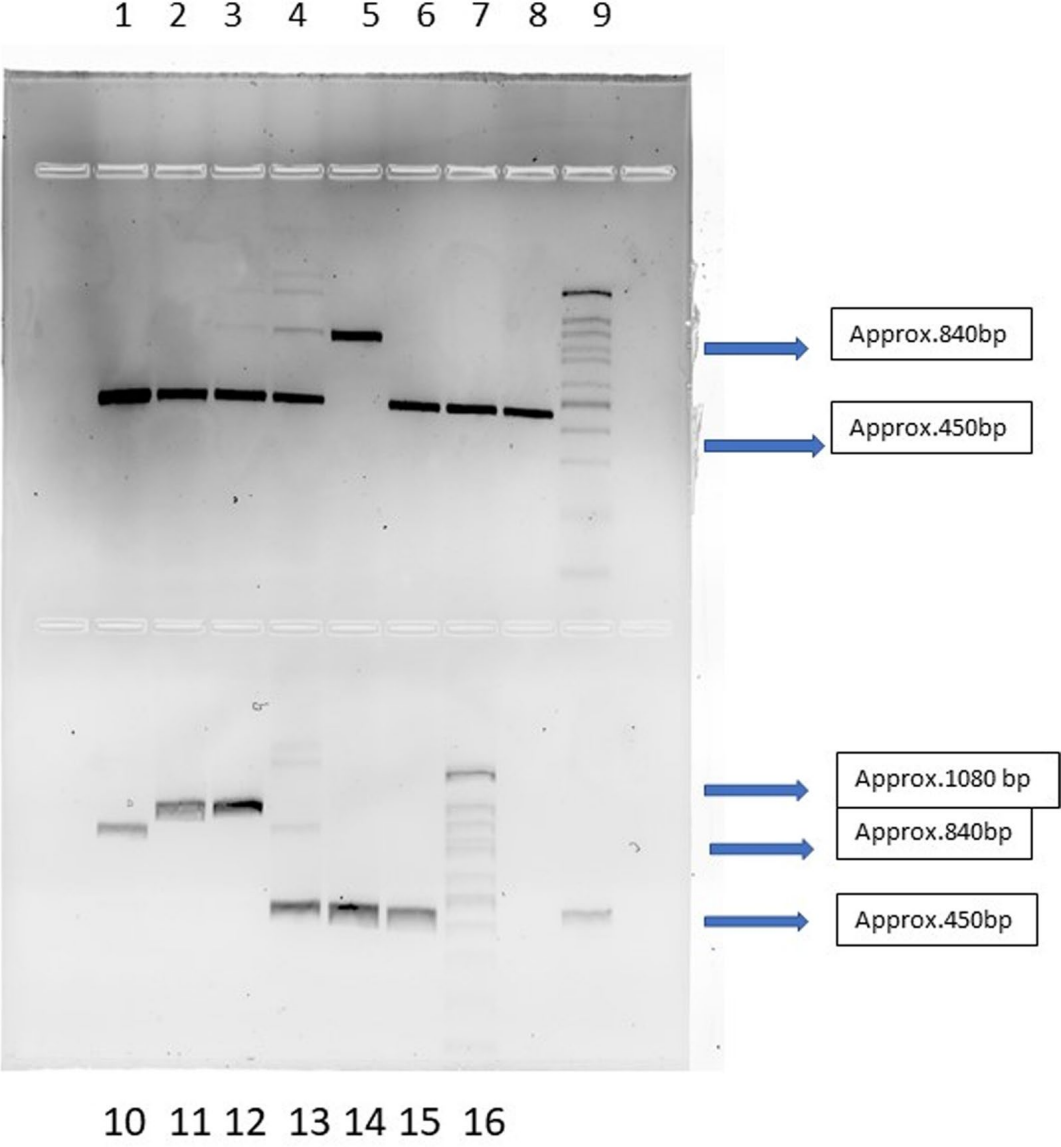
Agarose gel electrophoresis for the ABC typing of test and reference strains. †Upper row: Lane 1-U1, 2-U2, 3-U3, 4-U4, 5-F1, 6-F2, 7-F3, 8-F4, 9-100 bp DNA ladder. Bottom row: Lane 10-S1, 11-S2, 12-S3, 13-S4, 14-*C. parapsilosis* (ATCC 22019), 15-*C. albicans* (ATCC 90028), 16–100 bp DNA Ladder. ‡Note- UAE strains-U1, U2, U3 U4 Finland strains-F1, F2, F3, F4 Salivary strains-S1, S2, S3, S4

Considering all 12 isolates, Genotype A was the most prevalent with 66.6% (8 of 12 strains) followed by Type B and D with 16.6% prevalence (2 of 12 strains each). Type C genotype was not present amongst the 12 isolates (Fig. 1).

### Virulence attributes

#### Biofilm formation

Biofilms formed by the 12 isolates were assessed using the CV assay and the XTT assay at 48 h. The UAE and the Finland isolates were stronger biofilm formers when compared with the salivary isolates (Table 2). No significant association could be discerned between the genotypes of the strains and their biofilm forming ability.

**Table 2:** Comparison of virulence properties between the UAE strains, Finland strains and the saliva strains with reference strains; *C. albicans* (ATCC90028) and *C. parapsilosis* (ATCC 22019)

Strain	N	CV assay Mean (SD)	*p* value	XTT assay Mean (SD)	*p* value	Phospholipase Mean (SD)	*p* value	Haemolysin Mean (SD)	*p* value
*C. albicans* ATCC 90028	1	3.67(0.25)		1.43(0.34)		0.46(0.06)		0.51(0.02)	
*C. parapsilosis* ATCC 22019	1	ND		ND		1.00		1.00	
UAE^a,b^ *C. albicans*	4	2.69 (1.38)	0.69 NS	1.07(0.71)	0.74 NS	0.46(0.08)	0.035^c^	0.53(0.13)	0.023^c^
Finland^a^ *C. albicans*	4	2.94 (1.03)		0.81 (0.51)		0.64 (0.10)		0.56(0.01)	
Saliva^b^ *C. albicans*	4	2.29 (0.50)		0.83 (0.18)		0.61(0.07)		0.49(0.04)	

^a,^^c^Significant at *p* < 0.05 (ANOVA) for phospholipase

^b,^^c^Significant at *p* < 0.05 (ANOVA) for haemolysin

N, number of strains; ND, not done; NS, not significant

On comparison of CV and XTT readings of all the isolates, there was no significant difference in the readings of the biofilm forming ability indicating the concordance of these two methods of evaluation (*p* > 0.05).

#### Phospholipase activity (Pz)

The presence of phospholipase activity (Pz) was portrayed as a zone of white precipitation around the colony. All 12 isolates were phospholipase producers, and there was a significant difference in the phospholipase activity amongst the three groups with the UAE isolates showing superior phospholipase production capacity. Further, significant difference phospholipase activity was also observed between the UAE, and Finland endodontic isolates (*p* < 0.05; Table 2). No significant association could be discerned between the genotypes of the strains and their phospholipase activity.

#### Haemolysin activity (Hz)

All the tested isolates were strong haemolysin producers, and there was a significant difference in the haemolysin activity amongst the three groups with the salivary isolates showing superior activity compared to the endodontic isolates, (*p* < 0.05; Table 2). No significant association could be discerned between the genotypes of the strains and their haemolysin activity.

### Antifungal susceptibility

All isolates were sensitive to the four antifungals used, namely Amphotericin B, Fluoconazole, Ketoconazole and Nystatin. The values fell within the standard range of MIC values for the control isolates for *C. albicans* (ATCC 90028) and *C. parapsilosis* (ATCC 22019) as per the CLSI standards.

There was a significant difference in the MIC values of the three groups with fluoconazole (*p* = 0.036; Table 3). The salivary isolates were more sensitive to fluoconazole when compared to both of the endodontic groups. No significant associations could be made between the strain genotype and the antifungal sensitivity of all four antifungals.

**Table 3:** Comparison of antifungal susceptibility between the UAE strains, Finland strains and the saliva strains with reference strains *C. albicans* (ATCC90028) and *C. parapsilosis* (ATCC 22019)

Strain	N	Amphotericin B Mean (SD)	*p* value	Fluoconazole Mean (SD)	*p* value	Ketoconazole Mean (SD)	*p* value	Nystatin Mean (SD)	*p* value
*C. albicans* ATCC 90028	1	1.04 (0.36)		0.62 (0.00)		2.50 (0.00)		1.66 (0.72)	
*C. parapsilosis* ATCC 22019	1	0.41 (0.18)		1.67 (0.72)		0.26 (0.09)		0.62 (0.00)	
UAE^a^ *C. albicans*	4	1.09 (0.46)	0.051 NS	0.62 (0.17)	0.036^b^	1.41 (1.04)	0.947 NS	1.25 (0.33)	0.213 NS
Finland *C. albicans*	4	0.55 (0.10)		0.42 (0.15)		1.41 (0.79)		1.72 (0.59)	
Saliva^a^ *C. albicans*	4	0.65 (0.13)		0.34(0.05)		1.25 (0.34)		1.14 (0.36)	

^a,^^b^Significant at *p* < 0.05 (ANOVA) for Fluoconazole

N, number of strains; NS, not significant

## Discussion

A number of previous workers have reported the prevalence of *Candida* species in infected root canal systems [2, 14, 29, 30]. However, the prevalence rates appear to vary considerably in these reports. For instance, similar to our report, Siqueira et al. [31], noted fungi in only one of 50 (2%) primary root canal infections, whilst in another study, Lana et al. [29] noted 7.4% prevalence of *Candida* species in 31 patients with pulpal necrosis. Further, in a landmark study of 967 Finnish patients unresponsive to conventional treatment, Waltimo et al. [18] isolated *C. albicans* from 5.49% cases. On the contrary, Baumgartner et al. [30] detected *C. albicans* in 21% of their infected new cases. Ashraf et al. [34] in their study of post-treatment infected cases, could isolate *C. albicans* in four cases (13.3%) without periapical lesions and in eleven cases (36.7%) with periapical lesions using only routine culture methods. A study done by Anderson et al. [35] on 21 post-treatment cases using culture methods and culture independent methods revealed that fungi could be identified in just one sample (5%) using the universal fungal PCR. Dumani et al. [36] could identify *C. albicans* in 20% of necrotic teeth and in 11% retreated root canal infections, using polymerase chain reaction [36]. These disparate data have been recently systematically reviewed by Persoon et al. [32], and they concluded that the overall prevalence of fungi in the root canal systems is 7.5%, and *C. albicans* is the most frequently isolated species, a figure similar to our findings. This data may further imply that yeasts were present in higher proportions in post treatment infections compared to primary infections [33], although this finding was not backed by statistical evidence.

On genotyping the isolates, the majority of the strains in our study belonged to Type A (66%). Others have also confirmed a predominance of genotype A, in oral mucosal candidal infections [37]. Karahan et al. [38] studied the association between genotypes and invasiveness of *C. albicans* and showed that Genotype A was the most invasive sub-type, akin to blood stream isolates. All the UAE endodontic isolates and three of four Finland archival strains were genotype A, which could be one explanation for their invasiveness, and the ability to withstand the thermal, osmotic and oxidative stressors within the hostile root canal environment. However, we were unable to elicit an association between the genotype and the examined virulence attributes of the strains, as previously reported [13].

Studies have shown that the biofilm forming ability of *Candida* is linked to enhanced virulence and increased resistance to antifungals [39]. Hence we hypothesized that the root canal strains would be intrinsically good biofilm formers. However, we could not elicit such a difference between the endodontic, and the salivary groups as all were uniformly good biofilm formers.

Candidal phospholipases are thought to hydrolyze one or more ester linkages of glycerophospholipids and thereby degrade eukaryotic cell membranes leading to cell lysis and death facilitating yeast adherence and tissue penetration [25]. We noted that, the intracanal *C. albicans* isolates in UAE exhibited greater phospholipase activity compared to the salivary isolates. Similar observations have been reported by Pinto et al. [40] in *C. albicans* isolates from denture-related stomatitis.

Haemolysin, is another virulent attribute that helps *Candida* to survive within the host through acquisition of iron in the econiche [27]. All isolates in our study were strong haemolysin producers but the UAE isolates showed significantly stronger haemolytic activity relative to Finnish endodontic isolates. Interestingly, concurring with our findings, Gomes et al. [41], noted that *Candida albicans* from endodontic lesions in diabetics were more haemolytic than those from normoglycemic individuals.

In the root canal systems, it is likely that *C. albicans* exist in the biofilm rather than the planktonic phase cells. This biofilm lifestyle is known to better resist antifungal assault in comparison to their planktonic counterparts [9]. Nevertheless, we could not discern significant differences, in the antifungal sensitivities of nystatin, amphotericin B and ketoconazole, between the endodontic strains and the salivary strains, except for fluconazole where the salivary strains were more resistant to the latter. One reason for this may be the small number of strains evaluated in the study. Additionally, there was no significant association between the genotype of the isolates and the antifungal susceptibilities.

Although different irrigating solutions targeting yeasts have been in use by endodontic practitioners [43–45], candida biofilms have a unique ability to differentially tolerate endodontic irrigants. They are able to persist after treatment with 3% sodium hypochlorite (NaOCl) and regrow to levels that are comparable to untreated biofilms [46]. Ethylene di-amine tetra-acetic acid (EDTA) can significantly inhibit persistent NaOCl treated biofilms, though not completely, indicating possibilities for secondary endodontic infections. Irrigating root canals with higher concentrations of NaOCl and chlorhexidine are shown to have better antifungal effect on *C. albicans* when compared to even the antibiotic-based root canal irrigation solutions [45]. This study showed that three out of four isolates were found in post-treatment infections; therefore, it is recommended to use high concentrations of NaOCl in retreatment cases.

Our study has the following limitations. First, the relatively small number of endodontic isolates from the Middle East where the study was conducted. It should be mentioned that we evaluated over 70 patients with endodontic infections and only four root canals yielded yeasts. Second, we used conventional techniques to isolate and identify the yeasts. Clearly, the advent of next generation sequencing (NGS) platforms has significantly advanced mycobiome studies and elucidated the endodontic mycobiome as a rather complex ecosystem comprising both cultivable and non-cultivable fungi [32, 42].

## Conclusions

The prevalence of *C. albicans* in infected root canals of patients attending a dental hospital in UAE, was 5.6%. The strains isolated were good biofilm formers, possessed strong phospholipase and haemolysin activity and were mostly of the genotype A. No significant association could be found between the antifungal sensitivities and the genotypes, or the isolation niches. The fact that the root canal isolates possessed significant hydrolase activity, imply that they are equipped with virulence attributes necessary for survival within a harsh ecosystem.

## Data Availability

The data supporting the results reported in this manuscript can be provided upon request to the journal by the authors.

## Abbreviations

ANOVA: Analysis of variance
BHI: Brain–heart infusion
*C. albicans*: *Candida albicans*
*C. parapsilosis*: *Candida parapsilosis*
CV: Crystal violet
DMSO: Dimethylsulphoxide
EDTA: Ethylene di-amin tetra-acetic acid
FBS: Fetal bovine serum
Multiplex PCR: Multiplex polymerase chain reaction
OD: Optical density
ODc: Optical density cut-off
PA: Periapical
PBS: Phosphate buffered saline
RPMI: Roswell-Park Memorial Institute
SDA: Sabouraud dextrose agar
NaOCl: Sodium hypochlorite
SD: Standard deviations
XTT: (2,3-Bis[2-methoxy-4-nitro-5-sulfo-phenyl]-2H-tetrazolium-5-carboxanilide
